# Mechanically Enabled Two-Axis Ultrasonic-Assisted System for Ultra-Precision Machining

**DOI:** 10.3390/mi11050522

**Published:** 2020-05-20

**Authors:** Nan Yu, Jinghang Liu, Hélène Mainaud Durand, Fengzhou Fang

**Affiliations:** 1 Centre of Micro/Nano Manufacturing Technology (MNMT-Dublin), University College Dublin, D04 V1W8 Dublin, Ireland; nan.yu@ucd.ie (N.Y.); jinghang.liu@outlook.com (J.L.); 2 Survey, Mechatronics and Measurements Group, European Organization for Nuclear Research, 121 Geneva, Switzerland; Helene.Mainaud.Durand@cern.ch

**Keywords:** optical moulds, steel cutting, FEA, control system

## Abstract

With the use of ultrasonic-assisted diamond cutting, an optical surface finish can be achieved on hardened steel or even brittle materials such as glass and infrared materials. The proposed ultrasonic vibration cutting system includes an ultrasonic generator, horn, transducer, cutting tool and the fixture. This study is focused on the design of the ultrasonic vibration cutting system with a high vibration frequency and an optimized amplitude for hard and brittle materials, particularly for moulded steel. A two-dimensional vibration design is developed by means of the finite element analysis (FEA) model. A prototype of the system is manufactured for the test bench. An elliptical trajectory is created from this vibration system with amplitudes of micrometers in two directions. The optimization strategy is presented for the application development.

## 1. Introduction

Complex surfaces are widely demanded in various applications including optics, bioengineering, and advanced manufacturing [1,2,3]. Ultra-precision diamond cutting is normally used for the fabrication of complex surfaces because it allows a high degree of freedom for the structural design [4,5]. However, the diamond tool is not applicable for the cutting of ferrous materials, e.g., steel, due to the graphitization of the tool and massive tool wears in high pressure and temperature [6,7]. With the use of vibration-assisted machining (VAM), an optical surface finish can be achieved on steels, with significant advantages such as smaller cutting forces, longer tool life, and easier chip removal [6,8]. Reducing tool wear and improving the surface finish have been the key drive of the development of VAM in past decades [9,10,11]. A review on the wear mechanism and existing approaches for the tool wear reduction on diamond-cutting ferrous metals is conducted in [12]. Comprehensive review of VAM of ferrous and brittle materials is also introduced in [7,8], including one-dimensional (1D or linear) and two-dimensional (2D or elliptical [13]) vibrations. Ultrasonic vibration cutting (UVC) has been widely adopted for fabricating the optical steel moulds, as experiment reveals the diamond tool wear is effectively reduced [14,15]. Discontinuous contact takes place in the UVC process, so the cutting fluid could lubricate and cool the tool tip, then reduce the cutting temperature.

To increase the system efficiency and reduce the heat generation, a design with a resonant vibration is targeted in this study, which allows for the excitation direction of the supporting structure of the tool tip to be arranged as bending or longitudinal, depending on the relative movement between tool and workpiece. Bending vibration is referred to as the vibration in up-feed or cross-feed directions while longitudinal vibration is referred to as the vibration in the depth of cut direction. Generally, the transducer has three modes of vibration, i.e., longitudinal vibration, torsional vibration and bended vibration. Combined vibrational modes, such as the longitudinal–torsional, longitudinal–bended or bended–torsional vibration, will occur when the transducer has a unique structure. If the two directional vibrations have a phase shift of some degrees, the transducer will generate the elliptical vibration [16]. The research on 2D elliptical vibrator development mainly contains two ways of combination: (1) bending and longitudinal modes [9,17]; (2) Two bending modes in different directions [18,19]. The normal frequency of two bending modes vibrator is around 20 kHz [9,17] while a longitudinal and bending approach can be employed to achieve about 40 kHz [19]. In these experiments, the amplitudes of the cutting tips are mainly around 2–4 µm in the application on hard and brittle materials [17,18,19]. Surface roughness of the steel mould is reduced significantly by increasing the frequency from 40 kHz to 80 kHz [15]. Most recently, designs of ultrasonic vibration cutting devices have focused on increasing the operating frequency by typical commercial products, e.g., UTS2 (100 kHz) and ILSONIC (120 kHz), but with sacrificing the amplitude down to 0.1–2 µm [20,21,22]. Surface roughness of ferrous metal was achieved below 5 nm in Ra with these devices [23]. Meanwhile, a 3 DOF (degree of freedom) ultrasonic vibration tool for elliptical vibration cutting was developed for steel sculptured surface machining in [24,25]. The developed tool can be used to generate an arbitrary ultrasonic elliptical vibration in the 3D space so that it is suitable to machine the 3D sculptured surfaces.

Diamond cutting mechanisms for the fabrication of optical quality surface on hardened steel and brittle material by applying ultrasonic vibration have been intensively studied by Fang’s research group [26,27,28,29,30]. The ILSONIC device has been used for nanometric cutting of binderless tungsten carbide, with ultrasonic frequencies of 92.9 kHz and amplitudes of 1 μm and 0.5 μm in cutting and thrust directions [29]. UVC devices up to 65 kHz have also been developed in the past 10 years [26,27,28]. With the advantage of corrosion and wear resistance, high-quality mould steel is primarily used in the molding of precise parts. Fabrication of mould steels with optical quality still requires a polishing step after the UVC process. For the time being, UVC could be used to obtain surface roughness on mould steels in 4–10 nm in Ra. There is an urgent need to achieve an optical surface on moulds with 1 nm in Ra to remove the subsequent polishing. To meet this industrial challenge, a dedicated UVC system is designed and tested, with high operating frequency and without sacrificing the amplitude, for mould steel cutting applied in the intraocular lenses (IOLs) manufacturing chain [31].

## 2. Ultrasonic Vibration Cutting (UVC) System Design

**Figure 1:**
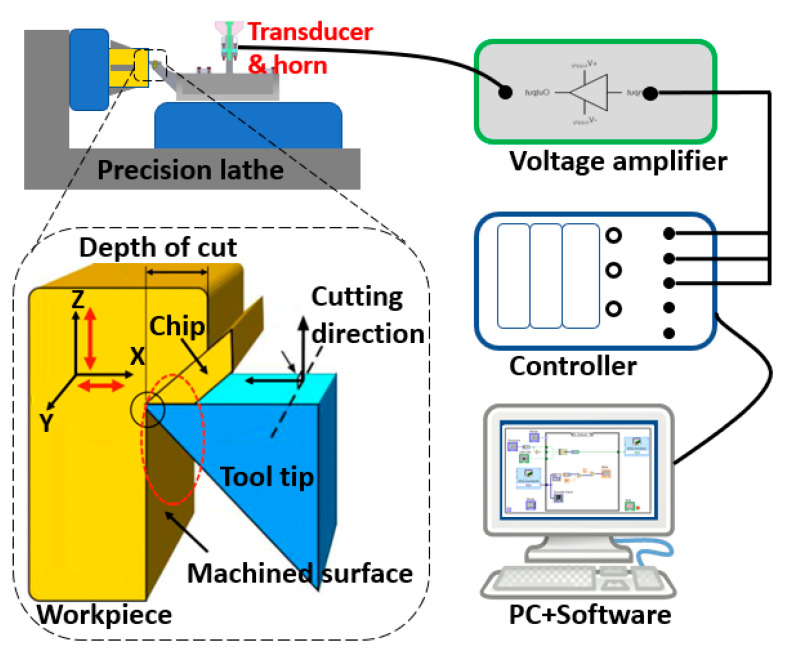
Schematic of the ultrasonic vibration cutting (UVC) system.

**Figure 2:**
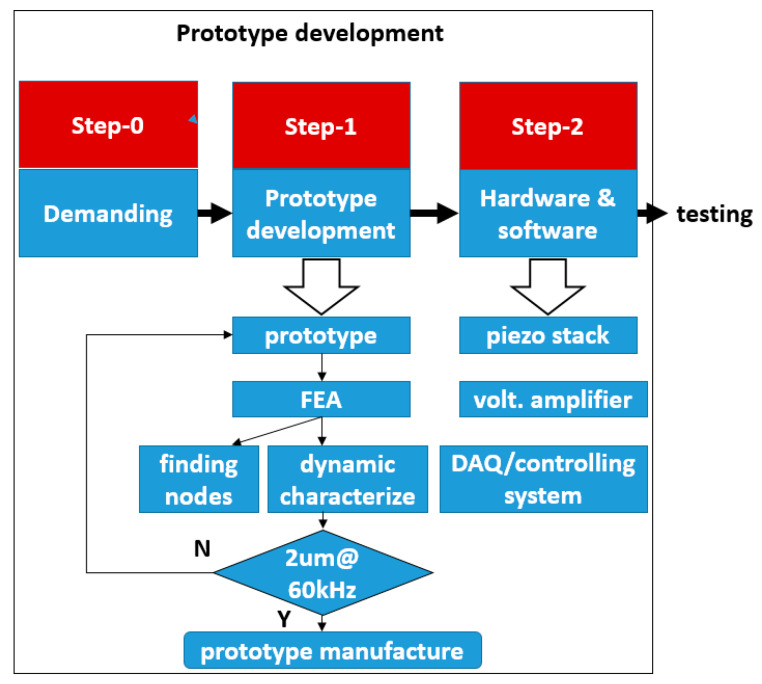
Development scheme of the proposed UVC system.

The proposed UVC system is demonstrated in Figure 1, including PC (user-machine interface, programming platform), controller (processing driving signal to oscillator and acquiring feedback data), oscillator (providing vibrations frequency ranging from a few Hz to thousands Hz), voltage amplifier (magnifying driving voltage from controller hardware to drive oscillator), transducer and horn assembly (amplifying the ultrasonic motion generated by oscillator). The sinusoidal signal is defined in the PC and software to set a given voltage and frequency, and the PZT (lead-zirconate-titanate ceramic) exciter generates the vibration when it receives the sinusoidal driving voltage. In this configuration, the vibration from the exciter is transferred and amplified by the cantilever-beam sonotrode into vibrations in two directions, and the tool tip on the lifted-up horn could oscillate in an elliptical trajectory. This lifted horn aims to increase the sagitta of the concave workpiece. This elliptical tool vibration in high frequency is expected to create better surface quality by reducing the cutting force significantly. The development of the prototype of the UVC system is schematically shown in Figure 2. 

**Figure 3:**
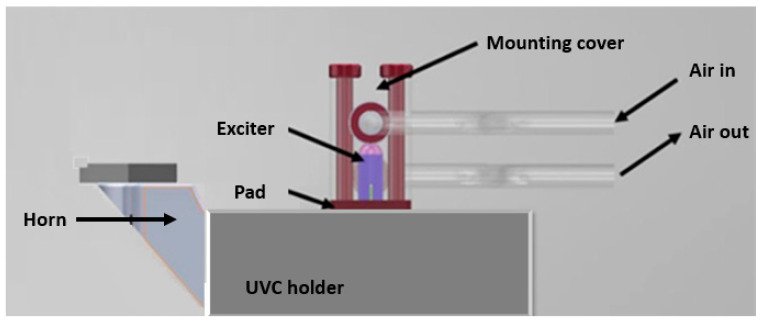
Design of the UVC system including a cooling arrangement.

The horn is expected to vibrate in 2 directions with a 1-D actuator at the resonant frequency. The inherent frequency of the horn is designed to ~100 kHz. The overall design of the UVC is shown in Figure 3. This design includes the horn, the tool, and the exciter with a cooling cover. Numerical analysis was carried out on both the horn and exciter with modal analysis and harmonic response analysis. The modal analysis of the horn aims to find the geometry determining the vibration frequency and amplitude of the tool, while the modal analysis of the exciter assembly aims to find the vibrations modes around 100 kHz. Harmonic response analysis of the horn will predict the tool characteristics during the oscillations.

In order to make an optical mould with freeform or structured surfaces, a cantilever structure of the horn is proposed, as it could avoid the mechanical collisions. Although some light materials, e.g., titanium alloys or aluminum alloys, have the advantages of high toughness and low temperature rise from internal loss heating, this also results in a low rigidity of the cantilever structure. Die steel with quenching heat treatment was applied to this project. As the fixation of the exciter part and the horn is critical, this was designed as a monolithic structure, increasing the coupling surface to cover the full width of the horn.

### 2.1. Horn Design

**Figure 4:**
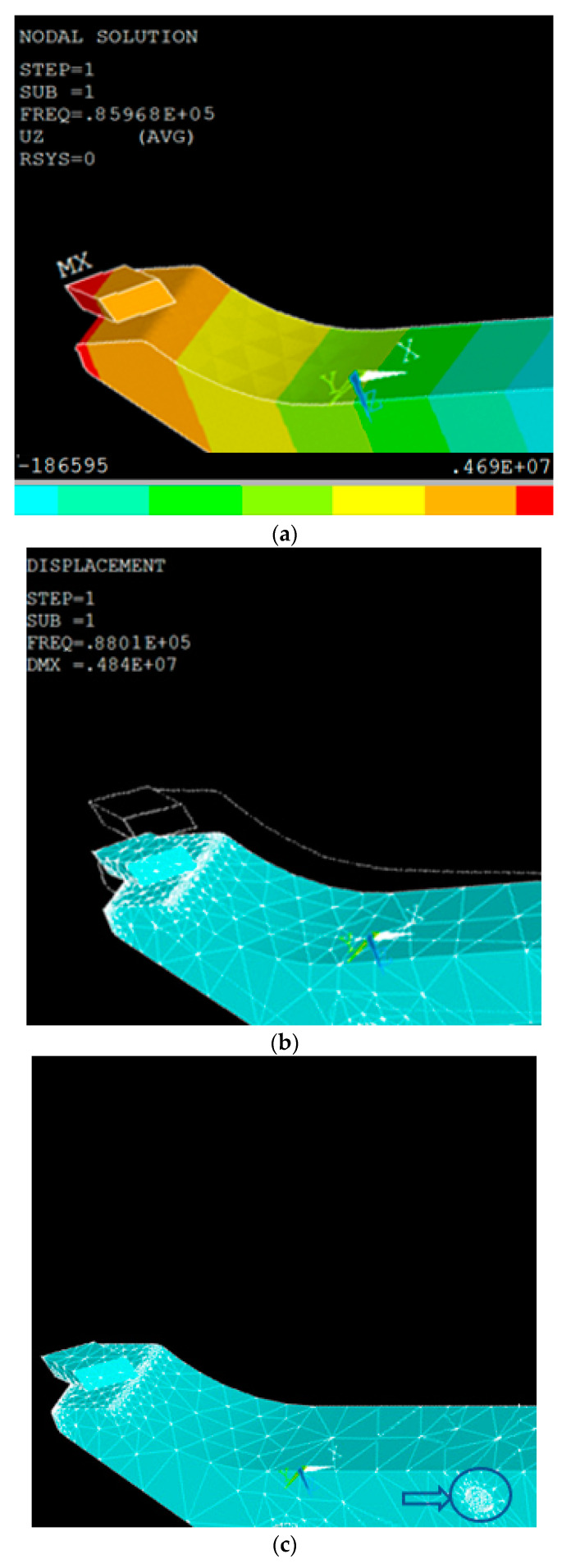
The modal analysis of the horn. (**a**) The amplitude in x direction; (**b**) The vibration in z direction; (**c**) The fixation position on the horn.

A finite element analysis (FEA) study was conducted through commercial software ANSYS APDL (Release 15.1, ANSYS Inc., Canonsburg, PA, USA) [32] for the modal analysis. As the material of the horn is steel, the property setting is: Young modulus *Ex* = 2.05 GPa, Density *ρ* = 7850 Kg/m^3^, and Poisson’s ratio PRXY = 0.27. The body element is Solid 185 and Meshtool is used to control the size of meshing (Figure 4b). Then the modal analysis is carried out with Subspace as the extraction method, to extract the modes of 10, with the range of 0 to 200 KHz. As this horn will transfer the vibration generated from the exciter to the diamond tool tip, the shape of the horn is optimized through a couple of iterations in order to find a maximum displacement of the diamond tool on this horn. A mathematical model based on Timoshenko beam theory was used to find the relationship between natural frequencies and the geometric parameters of the horn. Trial and error method was then applied for optimal design of the horn. The modal analysis enables the determination of the modes and nodal points on the cantilever beam. The calculated resonant frequencies in the range of desired frequency (100 ± 20 kHz) are fourth mode of longitudinal vibration at 86.968 kHz (Figure 4a) and sixth mode of bending vibration (Figure 4b) at 88.01 kHz. The difference of 1.04 kHz is acceptable for the further test. The fixation position is also confirmed based on the nodal points of the desired modes and marked in Figure 4c; the minimum displacement takes place around this position at a transversal vibration mode of 88 kHz.

### 2.2. Piezoelectric Exciter

The transducer of the UVC system contains a piezoelectric exciter and a seismic mass. A longitudinal vibration at the desired frequency (around 100 kHz) is generated from the exciter and transferred to the cantilever beam horn in the aforementioned way. Modal analysis of the transducer was conducted in FEA. The three natural frequencies (89.760, 100.546, 121.769 kHz) with longitudinal mode were found, and, the frequency of 90 kHz was selected for a better matching with the horn’s resonant frequency. The fixation position is also confirmed at the smallest displacement area at this vibration mode. Harmonic analysis of the transducer was carried out subsequently to find out the specific displacement value of the transducer at a given harmonic voltage.

The excitation of the transducer was generated by the piezoelectric actuator through the inverse piezoelectric effect. This PiezoDrive SA stack is a high-performance piezoelectric actuator with an ultraviolet (UV) cured epoxy coating for improved mechanical and humidity protection. The actuators (SA030305), data shown in Table 1 are matched to the range of PiezoDrive amplifiers and driver modules. To reduce mounting errors, a ceramic or stainless-steel ball end can be used to interface the stack actuator to the load. Flexural mechanisms are also applied to reduce bending moments during service.

To meet the frequency and amplitude requirements, higher resonant frequency of the selected piezo stack is preferred and ideally with a free-loading amplitude of 5–6 µm. There are mainly two challenges when using ultrasonic vibrating piezo stacks: quick heating up and high dynamic force. The quick heating problem can be overcome by using external cooling system as demonstrated in 2.3. Generally, 10% of the blocking force is recommended under static operation, while the maximum recommended preload under dynamic operation is 50% of the blocking force. However, due to a high dynamic vibration as in this project, a higher preloading force may be applied. The dynamic force generated by piezo component can be calculated by:

*F_dyn_* ≈ ±4π^2^*m_eff_* (∆L/2)f^2^ (1)

where, 

*F_dyn_*: Max. dynamic force, N;

*m_eff_*: Effective mass of the piezo stack actuator, kg;

∆*L*: Displacement (peak-to-peak), m;

*f*: Resonant frequency, kHz.

In this study, the displacement is calculated to be 1.42 µm in the cutting direction, the frequency is 100 kHz, so the dynamic force per kilogram is ±14.2 (kN/kg) as (2).

*F_dyn_*/*m_eff_ ≈* ∆*Lf*^2^*=* ± 1.42 × 10^−6^ × (100 × 10^3^)^2^ (2)

The flexure and horn parts are 40 g, so the dynamic force is calculated to be:

*F_dyn_ ≈* ± 14.2 × 0.04 = 568 N (3)

A preloading force higher than this value is needed to protect piezo stack from tensile force. Due to the limit of the amplifier bandwidth, the final vibrating amplitude is much smaller than 2 µm. Taking piezo stack SA030305 and amplifier PX200 as an example, the full voltage (150 V) amplitude of the piezo stack is 5.6 µm, so the amplitude under 90 kHz (20 V) is 0.7 µm, and the dynamic force is calculated as 223 N. 

### 2.3. Cooling System

Piezoelectric actuators dissipate heat when driven at full range with a high-frequency. PiezoDrive actuators can be operated continuously at temperatures up to 85 °C, whereas operation beyond this temperature may damage the actuator. Therefore, a gas cooling system is integrated, as shown in Figure 3. The flow rate is calculated from the following equations. For a sinewave, the power dissipation is shown in Equation (4) [33]:

*P* = π/4*fC*tan(*δ*)*U_p-p_*^2^ (4)

where,

tan(*δ*) = apparent dielectric dissipation factor = 0.12;

*C* = apparent PZT actuator capacitance (Farad) = 180 nF;

*U_p-p_* = peak-peak drive voltage (V) = 150 V;

*f* = operating frequency (Hz) = 100 kHz;

Considering the extreme situation for safety purposes, the maximum heat dissipated from the actuator has to be taken away by the air flow. According to the datasheet, the maximum temperature this actuator could bear is 150 °C, so given parameters of tan(*δ*) and *C* value as above. The maximum power dissipation (*P*) is calculated as 6.49 Watt, which will be removed by the 20 °C dry air flow. The mass flow rate (*V_c_*) can be calculated as 0.18 kg/h, by Equation (5)

*V_c_* = Δ*E_coolant_*/(*C_pc_*Δ*T*) (5)

where, 

Δ*E_coolant_* is the dissipated power calculated in Equation (4);

*C_pc_* = 1.003 kJ/kg·K is the specific heat capacity of air (up to 250 °C);

Δ*T* = 130 K = 150 °C − 20 °C.

### 2.4. Controlling System

A high-speed amplifier/piezo driver which can process 90 kHz driving voltage is needed in the proposed system. Accordingly, the bandwidth is the major parameter when choosing the amplifier. The power bandwidth is defined as the maximum frequency of an amplifier under full output voltage, while the small signal bandwidth (−3 dB gain bandwidth) is defined as the maximum frequency of an amplifier with a small signal applied, usually 200 mVp-p. When the amplifier output is open-circuit, the power bandwidth is limited by the slew-rate; however, with a capacitive load, the power bandwidth is influenced by the values of (1) output voltage range, (2) load capacitance, (3) output voltage (peak to peak), and (4) driving frequency. The authors did not find any existing commercially available amplifier with a power bandwidth up to 90 kHz. None of the selected amplifiers can process a full voltage with 90 kHz, and the final output frequency is a compromise between voltage range, piezo capacitance, and the driving voltage peak.

To meet the high-speed signal updating requirement, a PXI express chassis PXIe-1073 was selected to provide power, cooling, and a communication bus for following modular instruments and I/O modules. Waveform generator and multi-functional A/O were then selected to PXIe-5413 and PXIe-6361 (or PXIe-6363 with more A/O channels). These modules can be controlled by a controller PXIe-8301 that is remotely connected to an external PC. 

### 2.5. Harmonic Response Analysis of the UVC System

**Figure 5:**
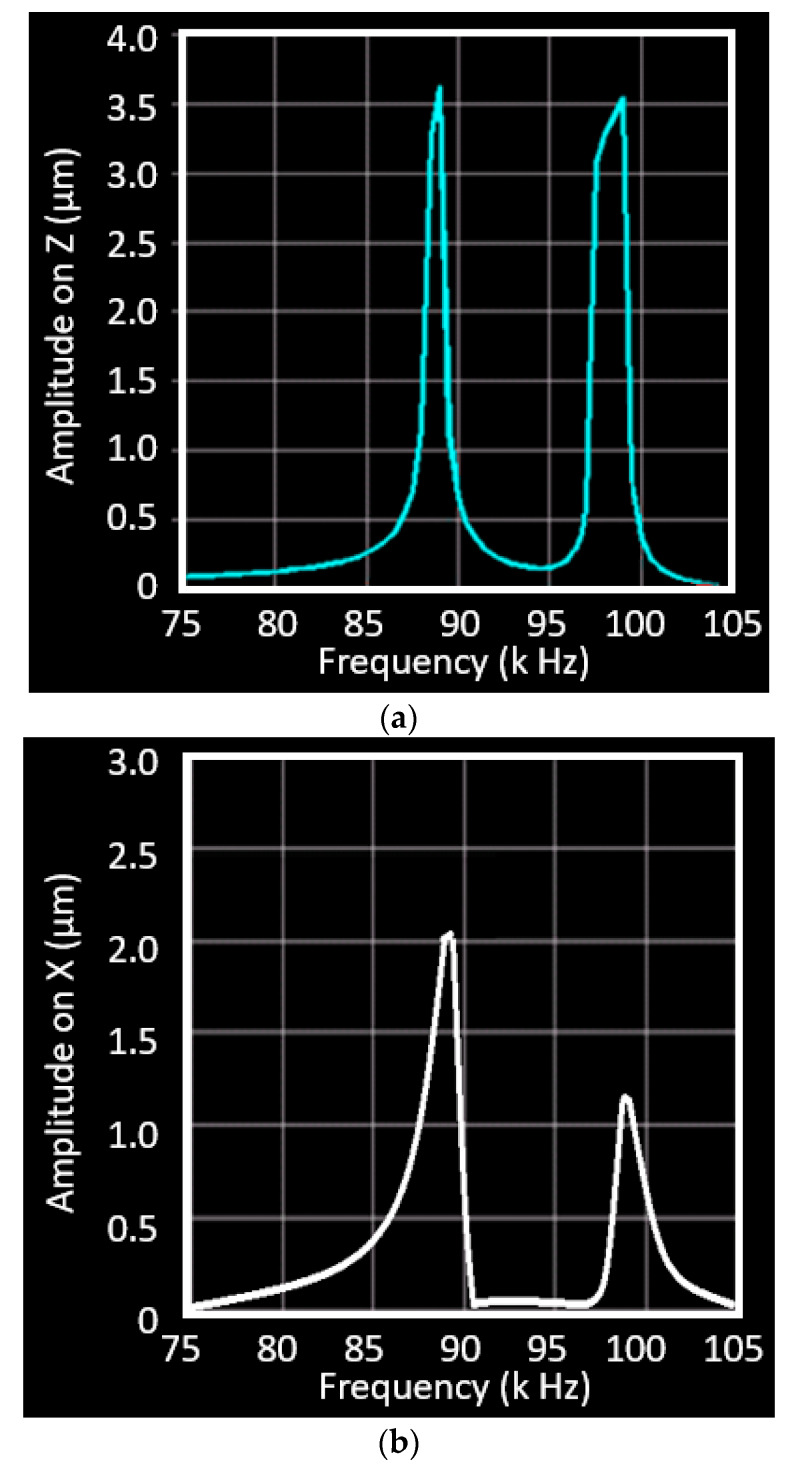
Harmonic response analysis of the UVC system: (**a**) the amplitude in z direction; (**b**) the vibration in x direction.

The harmonic response analysis was carried out in the FEA model to find the displacement of the horn in the given frequency range (around 90 kHz). The model creation and mesh generation are the same with that in the modal analysis. The coupled UVC system contains the horn, exciter, and a seismic mass on top of the PZT exciter. The exciter generates vibration, which is then amplified by the horn. The seismic mass has a function of impedance match to maximize the energy transfer from the PZT exciter to the loading tool tip on the horn. In this way, the amplitude of the vibration could be amplified as much as possible. According to the network theorem proposed for a high power transformer for impedance matching [34], the seismic mass of 80 g was tested in this analysis. The displacement of the fixation point is zero at the frequency of 90 kHz and the results of the tool tip are displayed in Figure 5. The Z-direction displacement of the tool tip reaches 3.55 µm at the frequency of 88.4 kHz and reaches the second peak of 3.53 µm at 98.2 kHz. The displacement of the tool tip in the X direction peaks at 89.3 kHz with amplitude of 2.0 µm, but is reduced to 1.2 µm in the next peak at 98 kHz. These results indicate that the vibration frequencies of the horn in two phases (Z and X directions) match with each other. This simulation will be verified by the displacement measurement of the vibrating tool in the next section.

## 3. Experimental Results and Discussion

**Figure 6:**
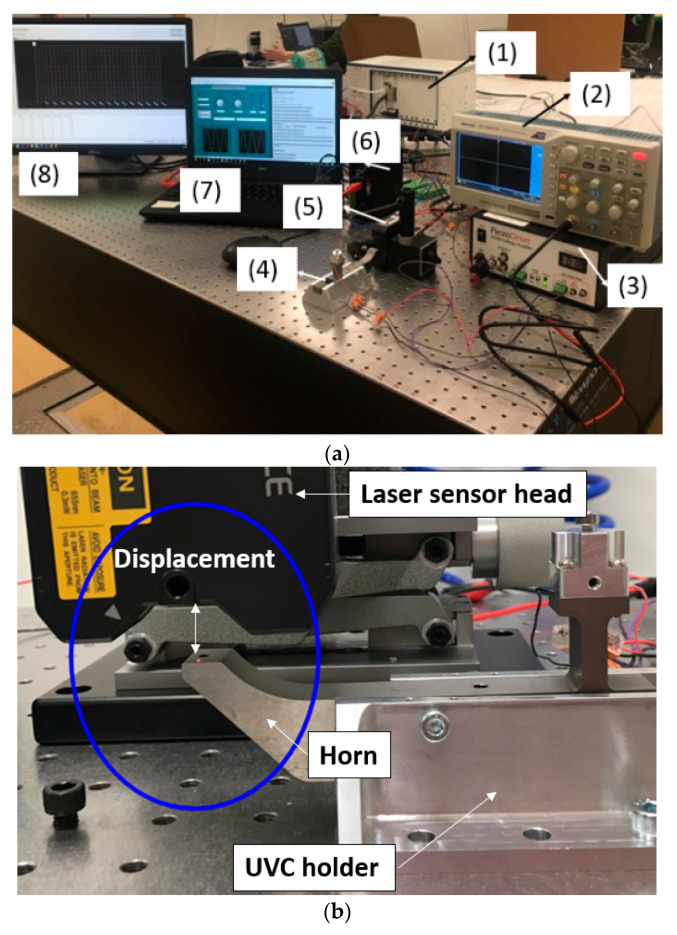
Offline test of the UVC system. (**a**) Experimental set-up on the optical table; (**b**) the displacement measurement in the vertical direction of the tool rest of the horn.

An experimental rig and control platform were developed to verify the feasibility of the proposed UVC system. The data needed were the displacement of the tool tip in horizontal and vertical directions. In this system (shown in Figure 6), piezoelectric stacks are employed as core oscillators due to their quick response, high acceleration, high accuracy and high stiffness. The following research aspects were addressed: piezo-stack dynamic response characterization; system resonant frequency identification; system nodal position determination and verification; and mechanical structure design and optimization. In the system, a high-resolution displacement sensor was used to monitor tip position and frequency. 

In Figure 6 the devices in this experimental set up include: (1) Controller for Data Acquisition (DAQ)and signal generation (National Instruments Corporation (UK) Ltd, Newbury, UK); (2) oscilloscope monitoring amplified voltage; (3) piezoelectric voltage amplifier (gain 20); (4) UVC horn fixed on isolation stage; (5) laser displacement sensor head; (6) laser sensor controller. (7) PC for signal generation; (8) monitor for laser signal sampling. The highest sampling frequency (392 kHz) of this sensor was used for the data acquisition with the repeatability of 0.1 µm.

**Figure 7:**
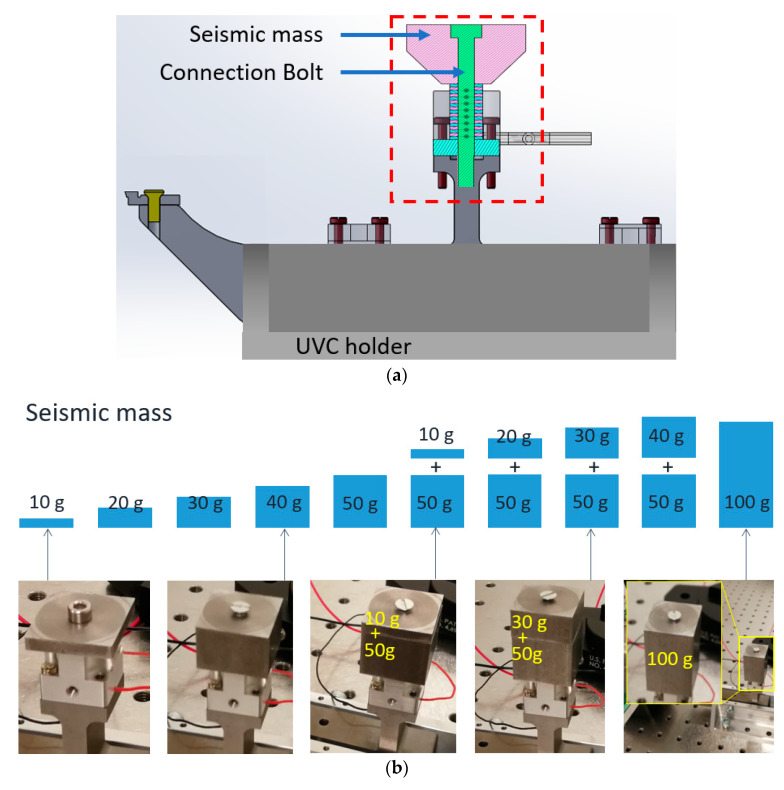
(**a**) Schematic of the vibration system including an adjustable seismic mass; (**b**) experimental measurements of the vertical displacement in a set of seismic mass.

The voltage of sinusoidal excitation signals for the actuator was 140 V*_p-p_*. Different seismic masses were tested as shown in Figure 7a, by adding them on the top of the PZT stack in the dynamic point of view. Measurements were carried out with a set of masses from 10 g to 100 g (Figure 7b). The resonating frequency was measured to 89 kHz, while the amplitude has significant improvement as the force could be transferred efficiently to the UVC horn. A large amount of data were processed for this experiment with 10 groups of seismic mass from 10 g to 100 g at an interval of 10 g, and 5 iterations in each group for the analysis of repeatability, with deviation of about 10%. Table 2 illustrates the amplitude of the tool tip in the *Z* axis (vertical direction) and *X* axis (longitudinal direction). The amplitude of vibration increases as the mass is added, and then reaches the peak in specific mass value: amplitude in the Z axis achieves peak-to-peak 3.0 µm with a 100 g mass, while the amplitude in *X* axis decreases from 2.0 µm with 50 g mass to 1.6 µm with 100 g. The deviations in 4 groups (60, 70, 80, 90 g) is two times higher than others (10, 20, 30, 40, 50, 100 g), because of the combination of two pieces of mass were used in the experiments (see Figure 7b). This is attributed to the multiple parts introducing decoupling vibration, although they are fixed tightly with screws. The vibration amplitude in two orthogonal directions, shown in Table 2, indicates that an elliptical trajectory of the tool tip is formed. This elliptical trajectory was created by this single PZT-driven UVC system with given mass weights (50–100 g), and the amplitude varied from 2.4 to 3.0 µm in Z direction and 2.0 to 1.6 µm in X direction. This experimental measurement of the tool tips’ displacement is in good agreement with the FEA simulation results, in which the Z direction is 0.5 µm (16%) higher and the X direction is 0.3 µm (15%) higher. Decoupling vibration is unavoidable for these connections among seismic mass, exciter, and horn. The resonance at the frequency of 89 kHz has a deviation of less than 1%.

## 4. Conclusions

A 2D ellipsoidal UVC system was achieved using a single driven vibration actuator. The developed prototype achieved micron amplitudes and ultrasonic frequencies. This UVC design was based on a longitudinal transducer and cantilever-beam horn with joint structure, that transfer the vertical vibration to vibrations in two directions. The design aimed to reduce the cutting force by creating a high-frequency elliptical vibration on the tool tip with a unidirectional PZT exciter. FEA modal analysis and harmonic response analysis were carried out to find the optimal vibration modes, frequencies and displacement at harmonic frequencies. The two resonance modes in longitudinal vibration (X direction) and bending vibration (Z direction) were coupled around 88 kHz. An experimental test of the UVC system with a set of seismic mass was carried out to measure the scale of the elliptical trajectory, and the voltage of a sinusoidal signal at 150 V was provided to the longitudinal PZT exciter. The UVC tool tip vibrated with amplitudes of around 3 µm in *Z* axis and 2 µm in *X* axis, when the input frequency reached 89 kHz. To further increase the frequency, the mechanical structure of the horn and the piezo-stack preload mechanism will be optimized in future work. The numerical calculation of tip displacement is within the range of the experimentally tested displacement, hence the FEA model has been proven for horn design and modification in the future. 

## Figures and Tables

**Table 1:** The specification of the piezo stack selected.

**Range +/− 10%**	**Length**	**Cross Section**	**Cap. +/− 20%**
5.6 µm	5 mm	3 × 3 mm	140 nF
**Mass**	**Blocking Force**	**Stiffness**	**Res. Freq.**
0.53 g	330 N	80 N/um	300 kHz

**Table 2:** Amplitude values in 10 groups of tests with seismic masses from 10 g to 100 g.

Seismic Mass (g)	Z (µm)	X (µm)
10	0.5 ± 0.1	0.9 ± 0.1
20	0.9 ± 0.1	1.3 ± 0.1
30	1.4 ± 0.1	1.7 ± 0.1
40	2.0 ± 0.1	1.9 ± 0.1
50	2.4 ± 0.1	2.0 ± 0.1
60	2.6 ± 0.2	1.8 ± 0.2
70	2.8 ± 0.2	1.8 ± 0.2
80	2.9 ± 0.2	1.7 ± 0.2
90	2.9 ± 0.2	1.6 ± 0.2
100	3.0 ± 0.1	1.6 ± 0.1
